# Awareness and intention-to-use of digital health applications, artificial intelligence and blockchain technology in breast cancer care

**DOI:** 10.3389/fmed.2024.1380940

**Published:** 2024-05-02

**Authors:** Sebastian Griewing, Johannes Knitza, Niklas Gremke, Markus Wallwiener, Uwe Wagner, Michael Lingenfelder, Sebastian Kuhn

**Affiliations:** ^1^Institute for Healthcare Management, Chair of General Business Administration, Philipps-University Marburg, Marburg, Germany; ^2^Institute for Digital Medicine, University Hospital Marburg, Philipps-University Marburg, Marburg, Germany; ^3^Department of Gynecology and Obstetrics, University Hospital Marburg, Philipps-University Marburg, Marburg, Germany; ^4^Commission for Digital Medicine, German Society for Gynecology and Obstetrics, Berlin, Germany; ^5^Department of Gynecology and Obstetrics, Martin-Luther University Halle-Wittenberg, Halle, Germany

**Keywords:** artificial intelligence, blockchain, digital health application, gynecology, breast cancer, oncology

## Abstract

Emerging digital technologies promise to improve breast cancer care, however lack of awareness among clinicians often prevents timely adoption. This study aims to investigate current awareness and intention-to-use of three technologies among breast cancer healthcare professionals (HCP): (1) digital health applications (DHA), (2) artificial intelligence (AI), and (3) blockchain technology (BC). A 22-item questionnaire was designed and administered before and after a 30 min educational presentation highlighting technology implementation examples. Technology awareness and intention-to-use were measured using 7-point Likert scales. Correlations between demographics, technology awareness, intention-to-use, and eHealth literacy (GR-eHEALS scale) were analyzed. 45 HCP completed the questionnaire, of whom 26 (57.8%) were female. Age ranged from 24 to 67 {mean age (SD): 44.93 ± 12.62}. Awareness was highest for DHA (68.9%) followed by AI (66.7%) and BC (24.4%). The presentation led to a non-significant increase of intention-to-use AI {5.37 (±1.81) to 5.83 (±1.64)}. HCPs´ intention-to-use BC after the presentation increased significantly {4.30 (±2.04) to 5.90 (±1.67), *p* < 0.01}. Mean accumulated score for GR-eHEALS averaged 33.04 (± 6.61). HCPs´ intended use of AI significantly correlated with eHealth literacy (ρ = 0.383; *p* < 0.01), intention-to-use BC (ρ = 0.591; *p* < 0.01) and participants´ age (ρ = −0.438; *p* < 0.01). This study demonstrates the effect that even a short practical presentation can have on HCPs´ intention-to-use emerging digital technologies. Training potential professional users should be addressed alongside the development of new information technologies and is crucial to increase HCPs´ corresponding awareness and intended use.

## 1 Introduction

Breast cancer is the most prevalent oncological entity affecting women in Germany, with over 70,000 new cases emerging each year (1). The adoption of digital technology in breast cancer care is becoming ever more essential due to the increasingly challenging care reality. While there was a short-lived decrease in cases in Germany thanks to improved screening processes, an aging population is likely to reverse this trend, resulting in more cases over time and increased demand for treatment. This challenge is intensified by the particularly favorable survival rate, which resulted from intensive research into innovative treatment options in the past decades, but simultaneously requires long-term follow-up adjuvant treatment to monitor recurrence and side effects, which demands ongoing commitment to patients (2, 3). On the other hand, persistent scientific efforts continue to uncover novel breast cancer treatment modalities. As a result, therapy options to treating breast cancer are rapidly evolving due to breakthroughs in diagnostic and treatment technologies. Recent diffusion of diagnostic tools, i.e., advanced genetic sequencing and the introduction of precision-targeted therapies including antibody-drug conjugates, are paving the way to the personalized cancer treatment approach (4, 5). This progress is accompanied with an overwhelming amount of complex data and information, which increasingly overwhelm practitioners in terms of complexity (6, 7).

In the meanwhile, the system of breast cancer treatment is confronted with an increasingly challenging care infrastructure (8). Sharing vital patient information, which is essential for making informed decisions in cancer treatment, is hindered by missing data infrastructure, interoperability and privacy issues (9). At the same time, the healthcare workforce is burdened by excessive documentation requirements and is shrinking due to demographic aging (10).

Innovative digital technologies can handle large-scale health data, uphold the corresponding privacy of patient data, and alleviate economic strains caused by increased patient numbers (11–13). According to a national study by the Commission for Digital Medicine of the German Society of Gynecology and Obstetrics (DGGG), the majority of gynecology specialists remain optimistic that digital advancements will alleviate respective challenges, enhance patient care, and foresee the integration of sophisticated algorithms (14).

These algorithms are commonly grounded in artificial intelligence (AI) techniques, including machine learning, deep learning, natural language processing, or neural networks. Although AI has become an integral part of daily life, it often stays unnoticed by its users. A recent UK study revealed that merely 17% of adults are cognizant of their engagement with AI technologies (15). In the realm of healthcare, AI applications have seen significant expansion in various domains such as image analysis, automated diagnostic procedures, intelligent drug delivery systems, and personalized treatment approaches (16). Specifically, AI technologies in breast cancer-related imaging, pathology, and supportive care not only alleviate the workload of healthcare professionals but also improve the accuracy and effectiveness in diagnosing and treating breast cancer (17).

Public awareness and understanding of emerging digital technologies vary. While over a third of the Germans is familiar and more than half feel confident in explaining AI, 38% have never heard of Blockchain (18).

Blockchain technology (BC), initially linked with the launch of the cryptocurrency Bitcoin in 2007, has rapidly evolved to encompass application areas extending beyond decentralized finance (19). Conceptually distinct from its role in payment networks, a BC functions as a distributed database, where data are stored across participating ledgers, eliminating the need for centralized storage (20). The adaptation of BC in healthcare has demonstrated its efficacy in providing efficient, decentralized data management, addressing existing data silos and high operational costs (21, 22). This approach was implemented in Estonia’s blockchain-based healthcare system infrastructure in 2008, showcasing the potential advantages in terms of cost and efficiency and has been transferred to diverse care settings, including breast cancer treatment (10, 22, 23). Drawing from these developments, the European Commission initiated regulatory actions in 2022 that aim to establish a European health data space, leveraging the full potential of decentralized data management for healthcare advancements in Europe (21).

In contrast to the more sophisticated functionality of AI and BC technology, digital health applications (DHA) have already found their way into German healthcare renumeration system. To leverage quick access to the benefits of digital medicine on clinical care, the “app on prescription” was introduced with the passing of the Digital Healthcare Act (Digitale-Versorgungs-Gesetz, DVG) in 2019, entitling over 70 million people with statutory health insurance receive reimbursement for DHA, called Digitale Gesundheitsanwendungen (DiGA). Up to now, 59 DHA have mastered the regulatory hurdles including three applications for breast cancer.

Nevertheless, in Germany, a cumbersome digitization process prevails and leads to an increasing gap between these evidenced clinical benefits of emerging digital technologies and their actual use, also in gynecological oncology (14, 24, 25). Their integration into healthcare settings rely on the attitudes and characteristics of both clinicians and patients for sustained awareness and intention-to-use (26, 27). Previous studies, i.e., on the integration of virtual reality in breast cancer treatment processes, underscore the importance of considering physicians’ psychological attitudes towards new technologies and exploring the transformative potential of interventions tailored to patients’ needs and outcomes (28, 29). To the best of our knowledge, the perception of emerging digital technologies, such as AI and BC, has not been investigated comprehensively within the context gyne-oncology.

This study seeks to evaluate healthcare professionals’ awareness and intention-to-use of these innovative technologies, and to determine if enhancing professional education could increase their intended use in breast cancer care. Furthermore, this research delves into the under-explored area of eHealth literacy among health professionals in gyne-oncology and its relation to both factors. As emerging technologies like AI and BC offer evidenced benefits for clinical care, it is essential to explore how professional education can bridge the gap between the potential and actual usage.

## 2 Materials and methods

### 2.1 Study design

The study was conducted as part of the regional gynecological specialist training days on breast cancer care (GYN-Fortbildungstage Mammakarzinom) in Marburg, Germany on December 6, 2023. The event encompassed presentations about current developments in breast cancer care, i.e., targeted therapies for breast cancer including antibody-drug conjugates or changes in reimbursable disease management programs for breast cancer patients. Only caregivers which were actively involved in patient care of the regional breast cancer care network of the county of Marburg-Biedenkopf did attend. Recruitment for the study was based on voluntary participation in the survey by the attendees of the mentioned event. An ethics vote was waived by the Research Ethics Committee of Philipps-University Marburg on November 13, 2023 (23-279 ANZ) with reference to the necessity of primary data anonymization, neglecting the option for follow-up survey. Participants who did not want to take part in the survey were able to leave the room beforehand. No one took advantage of this option, all attending participants in the event took part. The requirement for inclusion in the study was the active participation in the regional breast cancer care network and patient care. Exclusion was performed for participants who do not have a medical profession (e.g., administrative employees). The comprehensive survey was initially shared with attendees via a QR-code, with responses gathered directly through Google Forms (Google LLC, Mountain View, United States). Following the survey, participants underwent a 30 min educational presentation about the application of artificial intelligence and blockchain in managing breast cancer. The presentation included the demonstration of previously publicized concepts on combined application of artificial intelligence, i.e., the use of large language models as an adjunct for tumor boards, as well as blockchain technology, i.e., as technological framework for decentralized interoperable data management in care networks (8, 10, 30). After the presentation, two questions concerning the intended use of these technologies were posed once more.

### 2.2 Study population

The study encompassed physician and non-physician healthcare professionals involved in breast cancer care within the regional network under investigation. The preliminary set of questions aimed to gather fundamental demographic data, including age, gender, and details concerning their practice in either outpatient or inpatient settings, in addition to their level of professional training.

### 2.3 Questionnaire

The initial survey consisted of 22 items (see Supplementary material S1 for original German version), beginning with four questions to capture basic demographic details of the participants as described in 2.2. This was followed by a series of 10 questions evaluating the participants’ awareness and intention-to-use of three technologies (HCP): (1) digital health applications (DHA), (2) artificial intelligence (AI), and (3) blockchain technology (BC). Technology awareness was measured in binominal manner (yes or no) while intention-to-use was assessed using a 7-point Likert scale (1 = strongly disagree; 7 = strongly agree). The survey then addressed the components of the German version of the eHealth Literacy Scale (GR-eHEALS 1–8) (31, 32). Table 1 is provided to summarize the questionnaire and the abbreviations of its items.

**Table 1:** Questionnaire items.

Questionnaire part	Item	Question	Measurement
Demographics of study population	Age	How old are you?	Age in years
	Gender	Which gender do you consider yourself to be?	Single choice (Female, Male or Diverse)
	Sector	Do you work primarily in inpatient or outpatient care?	Single choice (Inpatient or Outpatient)
	Profession	In which position do you work?	Single choice (Other Medical Professional (No Physician), Final Year Medical Student, Resident Physician, Specialist Physician, Attending Physician, Chief Physician)
Digital health applications	DiGA1	I know digital health applications (DHA).	Single choice (yes or no)
	DiGA2	As a doctor, I have already prescribed digital health applications (DHA).	Single choice (yes or no)
Artificial intelligence	AI_1	I am aware of applications of artificial intelligence.	Single choice (yes or no)
	AI_2	I am aware of applications of artificial intelligence in healthcare.	Single choice (yes or no)
	AI_3	I am aware of applications of artificial intelligence breast cancer care.	Single choice (yes or no)
	ITU_AI	I would use applications of artificial intelligence in breast cancer care.	7-point Likert scale
Blockchain technology	BC_1	I am aware of applications of blockchain technology.	Single choice (yes or no)
	BC_2	I am aware of applications of blockchain technology in healthcare.	Single choice (yes or no)
	BC_3	I am aware of applications of blockchain technology in breast cancer care.	Single choice (yes or no)
	ITU_BC	I would use applications of blockchain technology in breast cancer care.	7-point Likert scale
GR-eHEALS	GR_eHEALS_1	I know how to find helpful health resources on the Internet.	5-point Likert scale
	GR_eHEALS_2	I know how to use the Internet to answer my health questions.	5-point Likert scale
	GR_eHEALS_3	I know what health resources are available on the Internet.	5-point Likert scale
	GR_eHEALS_4	I know where to find helpful health resources on the Internet.	5-point Likert scale
	GR_eHEALS_5	I know how to use the health information I find on the Internet to help me.	5-point Likert scale
	GR_eHEALS_6	I have the skills I need to evaluate the health resources I find on the Internet.	5-point Likert scale
	GR_eHEALS_7	I can tell high quality from low quality health resources on the Internet.	5-point Likert scale
	GR_eHEALS_8	I feel confident in using information from the Internet to make health decisions.	5-point Likert scale

### 2.4 eHealth literacy scale

The eHealth Literacy Scale (eHEALS), originally introduced by Norman and Skinner in 2006, has since been adapted into various languages and validated across multiple settings (32). The 8-item scale is used for assessing electronic health literacy in research populations, employing a 5-point Likert scale ranging from 1 (strongly disagree) to 5 (strongly agree), with total scores spanning from 8 to 40. Higher scores denote greater literacy levels. In our study, we utilized the validated German version of the eHEALS (GR-eHEALS) by Marsall et al. (31). This study marks the first application of GR-eHEALS among health professionals in the field of gynecology, prompting us to examine reliability and validity in this novel context in line with Norman and Skinner. Internal consistency was measured through Cronbach’s alpha. Validity was examined using exploratory factor analysis. We employed the maximum likelihood method, adopting factors with an eigenvalue exceeding 1, in line with the Kaiser criterion. The decision on the number of factors to retain was based on the analysis of a scree plot.

### 2.5 Data analysis

Data analysis was conducted in IBM SPSS Statistics (Version 29.0.2.0 (20); IBM Corporation, Armonk, United States). We computed Spearman’s rho to establish nonparametric correlations for the variables of age, ITU_AI, ITU_BC, and eHEALS_Total, and these were then evaluated for their statistical two-tailed significance. Additionally, we carried out Mann–Whitney Test to compare ITU_AI and ITU_BC scores across binary categories of gender and sector.

### 2.6 Pre-and post-comparison of intention-to-use

Following the educational session, the intention-to-use query was repeated for the investigated technologies (ITU_AI; ITU_BC). Differences in pre-and post-comparison were assessed by conducting a two-tailed Sign test on the two variable pairings (ITU_AI_PRE 🡢 ITU_AI_POST; ITU_BC_PRE 🡢 ITU_BC_POST).

## 3 Results

### 3.1 Demographics

**Figure 1:**
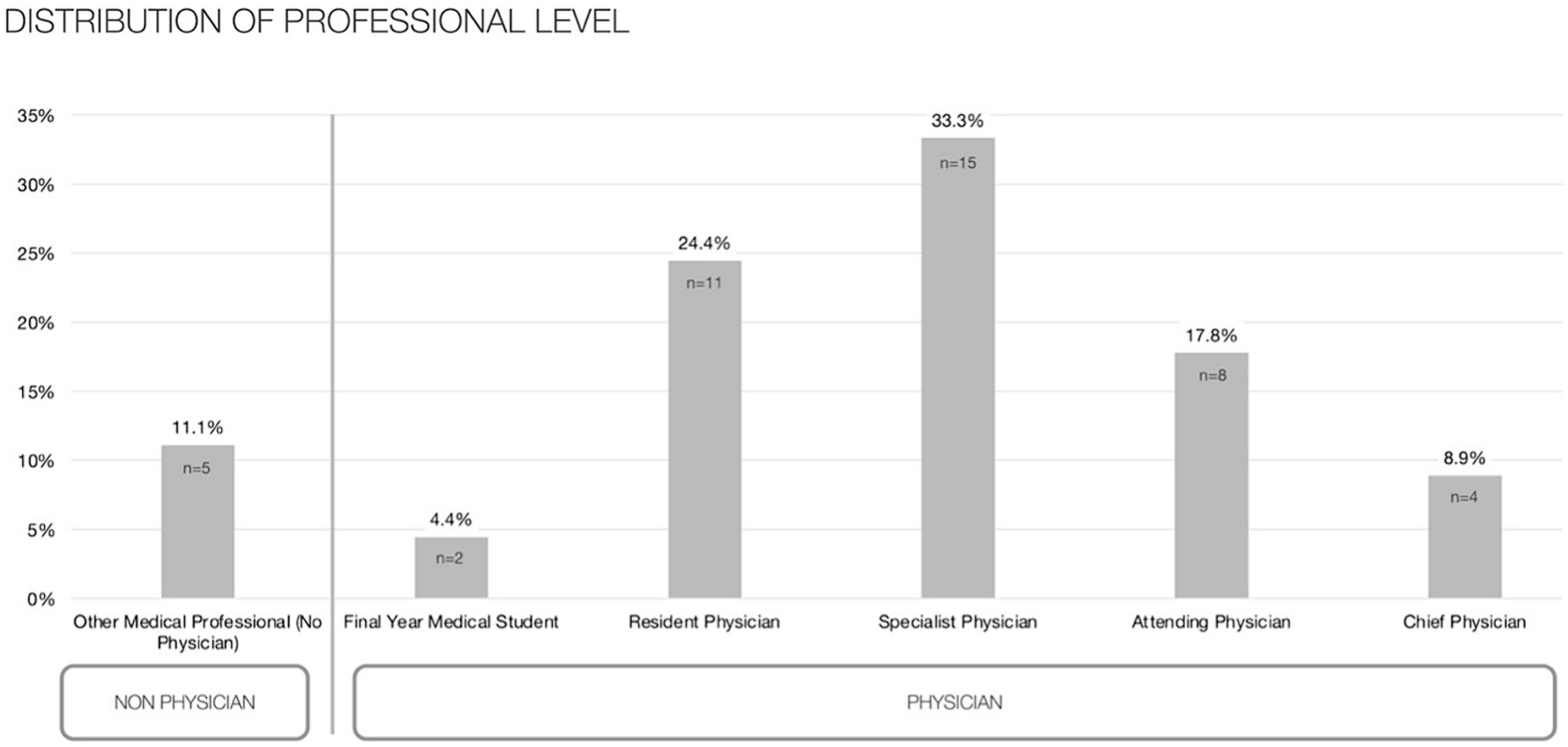
level of professional training of the study population.

The study population is characterized by a gender distribution of 57.8% female {mean age (SD): 48.26 (±13.96)} and 42.2% male {42.50 (±11.20)} for 45 participants {44.93 (±12.62), minimum 24 to maximum 67 years}. In terms of sectoral engagement, 35.6% are predominantly involved in outpatient care, while a majority of 64.4% operates within an inpatient setting. With respect to professional qualifications, the cohort comprises one-third specialists, accompanied by 24.4% of doctors in residency and 17.8% serving as attending physicians (see Figure 1).

### 3.2 Technology awareness

**Figure 2:**
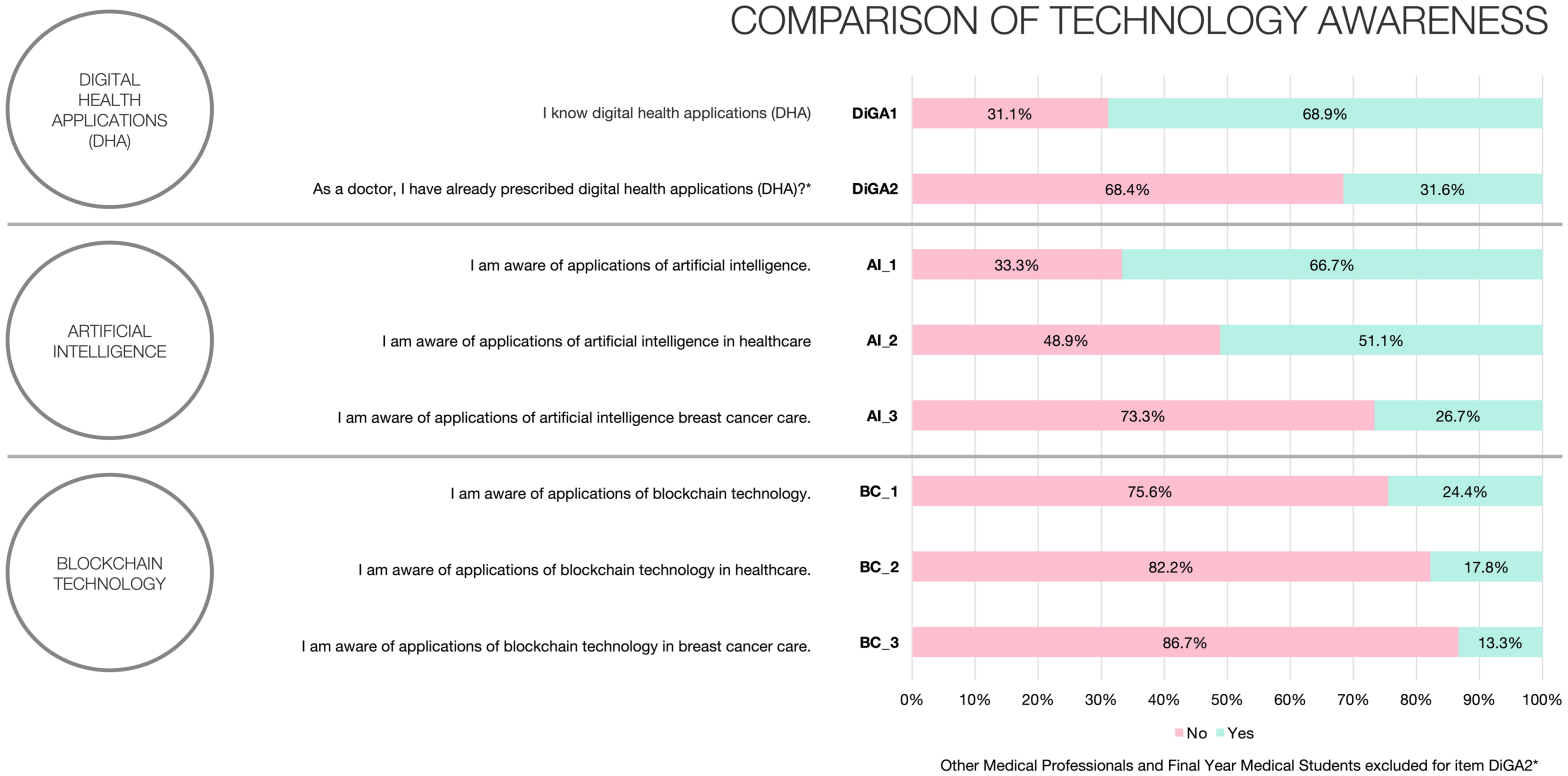
Technology awareness.

Figure 2 illustrates the reported awareness that participants have with the technologies being examined. DHA (Digitale Gesundheitsanwendungen, DiGAs) were the most recognized, with 68.9% awareness, followed closely by artificial intelligence at 66.7%, while blockchain technology as the least known, with only 24.4% of participants being aware of it. Regarding the specific utilization of these technologies in the healthcare sector or in the context of breast cancer treatment, the awareness rates were 51.1 and 26.7% for AI, and for blockchain technology, the rates were 17.8 and 13.3%, respectively.

### 3.3 Intention-to-use

**Figure 3:**
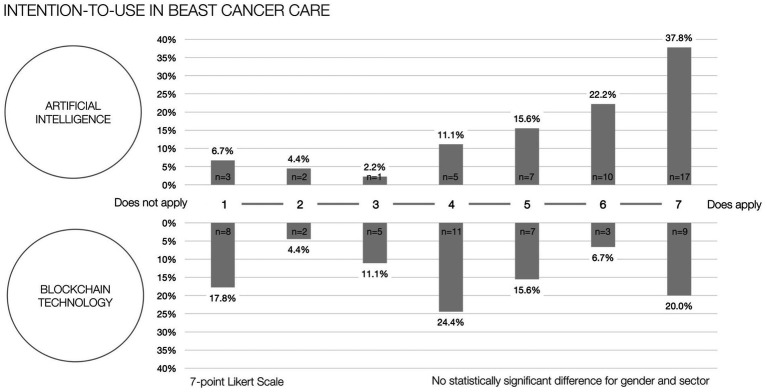
Intention-to-use in breast cancer care.

The initial intention-to-use, as measured by a 7-point Likert scale, yielded a mean of 5.42 (SD; ±1.82) for artificial intelligence and 4.16 (±2.04) for blockchain technology. Figure 3 depicts the distribution frequencies corresponding to the respective scores and types of technology. Moreover, the analysis did not reveal any statistically significant variances in the intention-to-use when comparing across the binary classifications of gender and sector for either technology.

### 3.4 Correlation matrix

Spearman’s rank correlation coefficient (ρ) was employed to evaluate the significance of associations among the variables: age, intention-to-use artificial intelligence (ITU_AI), intention-to-use blockchain technology (ITU_BC), and the aggregate scores of the German eHealth Literacy Scale (GR_eHEALS_Total). Consistent with the thresholds defined by Cohen et al., a robust positive correlation was identified between ITU_AI and ITU_BC (ρ = 0.591; *p* < 0.01) (33). Concomitantly, an intermediate-level negative correlation was discerned between participant age and ITU_AI (ρ = −0.438; *p* < 0.01), while an intermediate-level positive correlation was found between ITU_AI and GR_eHEALS_Total (ρ = 0.383; *p* < 0.01) (Table 2).

**Table 2:** Correlation matrix.

Correlation Matrix (Spearman’s rho, ρ)				
	Age	ITU_AI	ITU_BC	GR_eHEALS_Total
Age	1.000	−0.438**	−0.099	−0.125
ITU_AI	−0.438**	1.000	0.591**	0.383**
ITU_BC	−0.099	0.591**	1.000	0.206
GR_eHEALS_Total	−0.125	0.383**	0.206	1.000

** Correlation is significant at the 0.01 level (2-tailed). Bold values represents significant correlation coefficients.

### 3.5 eHealth literacy scale

For the exploratory factor analysis, significant findings from Bartlett’s test of sphericity (χ 2 = 304.000, *p* < 0.001) supported the factorability of the correlation matrix. The high value of Kaiser-Meyer-Olkin test (0.848) showed adequate sampling. Maximum likelihood method confirmed a single factor, which emerged from an initial eigenvalue of 5.47 and explained 68.37% of the total variance explained for. The factor loadings for this model were between 0.619 and 0.906. The data yielded a mean accumulated score for GR-eHEALS of 33.04 (SD ± 6.61; 95% CI {31.06, 35.03}) with a calculated Cronbach’s alpha amounting to 0.928. Detailed results of the exploratory factor analysis and the corresponding scree plot are presented in Supplementary material S2, S3.

### 3.6 Change in intention-to-use after educational presentation

**Figure 4:**
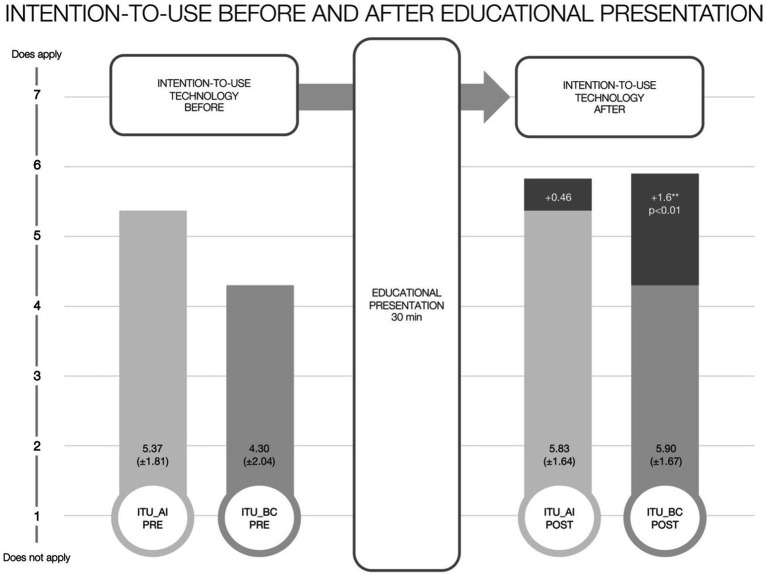
Change in intention-to-use before and after educational presentation.

With regard to the post-education survey conducted, 30 respondents repeated the question on intention-to-use AI or BC on a 7-point Likert scale. In this sub-group, the mean intention-to-use AI was 5.37 (±1.81) before the session and 5.83 (±1.64) afterwards, which did not show a statistically significant change. The intended usage of BC did significantly increase from an initial average of 4.30 (±2.04) to 5.90 (±1.67), marking an improvement of +1.6 (*p* < 0.01) (Figure 4).

## 4 Discussion

Germany’s healthcare system is characterized by a mandatory health insurance model, with the majority of citizens (89%) covered under statutory health insurance and a smaller fraction (11%) opting out for private health insurance (34). This system ensures extensive health coverage and contributes to the country’s high healthcare spending of 12.9% of its Gross Domestic Product (GDP) in 2021, surpassing the European Union (EU) average by two percentage points (34).

The nation offers a substantial hospital sector, evident in its high number of hospital beds, ranking second in the EU behind Bulgaria and considerably above the EU average. Germany’s physician and nurse density has increased at higher pace and surpasses European standards, reflecting a commitment to accessible medical care (34). According to the EU Statistics on Income and Living Conditions (EU-SILC) survey, the proportion of reported unmet medical needs in Germany due to costs, waiting times or distances is among the lowest in the EU without differences between income groups (35). The country exceeds OECD averages in life expectancy, preventable mortality, and healthcare coverage, with a notable 85% of the population expressing satisfaction with healthcare quality (34).

Despite these strong indicators, the country reveals a rather low performance in the adoption and implementation of digital health solutions (36). Despite the assured reimbursement of telemedicine, Germany, together with its neighboring country France, has the lowest proportion of remote medical consultations in the EU (34). The OECD Digital Health at a Glance 2023 report explores the concept of digital health readiness by evaluating their member countries´ policy, analytical, technical and social environment in terms of twelve digital health readiness indicators, which should enable the successful use of digital health. Denmark distinguishes itself as a consistent leader, topping seven out of twelve indicators. Other countries like Finland, Korea, Sweden, Japan, the United States, and the Netherlands also showcase significant achievements. In comparison, Germany clearly lags behind, only performing well with respect to two OECD indicators, namely by offering a national strategy for digital health and high levels of digital security (36).

### 4.1 Principal findings

Public awareness and understanding of emerging information technologies vary and their application often stays unnoticed (15, 18, 37). Despite evidenced benefits for clinical care, a gap between the digital technologies´ potential and actual implementation persists (24). The dissemination of innovative technologies in healthcare necessitates an examination of how professional training can help to close this gap. A study conducted by the Commission for Digital Medicine of the German Society for Gynecology and Obstetrics underlines a significant incongruity between the perceived benefits of digital medicine and its practical application in gynecological care in Germany (14). Despite 78.4% of gynecologists recognizing the ability of digital medicine to reduce their increasing workload, only 13.5% acknowledge receiving institutional training. The research identifies critical obstacles to the adoption of digital medical technologies, primarily grounded in the scarcity of time and a deficiency in knowledge. This study illustrates that even a short training session of thirty minutes can significantly increase the intention-to-use less-known technologies like blockchain, elevating its intended use to the same level as that of more familiar technologies such as artificial intelligence.

### 4.2 eHealth literacy of gyne-oncological health professionals

eHealth literacy refers to the capacity of individuals to use emerging information and communications technologies for the enhancement or facilitation of health and healthcare services (38). Previous research has established the eHEALS as a reliable and valid measure of eHealth literacy across different contexts (32). This study represents the first utilization of the German version of eHEALS (GR-eHEALS) to delineate the eHealth literacy of healthcare professionals in gynecological oncology, affirming the tool’s applicability in this context (31). Additionally, the positive correlation between eHealth literacy scores and the intended use of AI reinforces the tool’s validity in assessing competencies relevant to digital medicine. Our findings align with past research, which found a positive correlation of eHEALS with internet usage and reported computer knowledge (38, 39). The study confirms a sufficient degree of eHealth literacy among the investigated gyne-oncological care professionals (31, 32). This suggests that healthcare professionals in breast cancer care possess substantial digital skills to use emerging information technologies.

### 4.3 Differences in technology awareness

Despite demonstrating a satisfactory level of e Health literacy, adoption rates of emerging technologies often fall short of expectations (14, 24). In a survey conducted by the German Society of Gynecology and Obstetrics, gynecological professionals were asked about their actual use of DHA (Digitale Gesundheitsanwendung, DiGA) and a mere 10.2% of gynecologists had ever prescribed an app (14). Nearly half of the questioned professionals were not even aware that these applications were available for prescription, despite them being reimbursable in Germany since 2019.

This study suggests that the prescription rate of DHA among physicians has increased and provides further insights on less common emerging information technologies of AI and BC. As such their awareness in healthcare and breast cancer care fall behind the DHA. Despite the growing body of evidence underscoring their clinical applicability, lower levels of awareness among health professionals regarding their use in the management of breast cancer persist.

### 4.4 Differences in intention-to-use

These differences are not only observable in the context of technology awareness but are particularly pronounced in their delineated intended use for breast cancer care. AI is more widely recognized and has a higher intended use for breast cancer care in comparison to BC. Furthermore, the analysis reveals that younger individuals or those with higher levels of eHealth literacy show a greater inclination towards using AI – an observation that seems to be intuitively logic at first glance. Nevertheless, these observations are not consistently applicable to the less known technology of Blockchain. Professional education of 30 min did boost the intention-to-use both AI and BC, with the impact being particularly noteworthy and significant in the case of BC. As a result, after the educational presentation the intended adoption of BC equalizes with the level of intended adoption of AI in breast cancer care. The incorporation of AI in healthcare helps with diagnostic support, patient interaction and treatment customization. The effectiveness of diagnosis and treatment is usually the focus of scientific analysis. However, for a successful integration of the technology into clinical practice, psychological and social issues such as trust in AI, dependency risks and changes in the doctor-patient relationship must also be considered as suggested by Triberti et al. (40). The research by Strika et al. and Sebri et al. highlights the importance of understanding the attitudes of healthcare professionals and patients towards AI, an aspect that is often overlooked in research (27, 29). They argue that future studies should examine the broader impact of AI on the social and organizational aspects of healthcare, not just its effectiveness in diagnosis and treatment.

### 4.5 Limitations and research perspective

The research utilized a single-center approach, limiting the wider applicability and relevance of its findings beyond the context of the German healthcare system and its peculiarities in regulatory and socioeconomic dimensions. Unlike Germany, which has established the DiGA process to manage the prerequisites for DHAs´ reimbursability, other European nations do not offer standardized frameworks. As such, the equivalent term of DiGA for a DHA in Germany, needs to be interpreted in a different light compared to other European partner countries.

In a national context, the study’s focus on a single region could be expanded to include multiple and diverse regions across Germany to enhance the national relevance of the findings. Nevertheless, this study, conducted during regional training on breast cancer care in Hesse, ensures a representative sample by interviewing a balanced mix of participants. The study’s demographic balance across gender and professional training, coupled with the high centralization of care and the socioeconomically balanced, predominantly rural area of Marburg-Biedenkopf county, mirrors German standards well enough to provide a solid foundation for this kind of health services research. Previous epidemiological studies of the care network have proven to capture German standards to a sufficient degree (8, 41).

Nevertheless, to improve the external validity and broader applicability of the results, future research should adopt a national multi-network and international approach for comparison. This would enable the study’s findings to be more transferable and applicable to various health systems and care settings. Furthermore, the present study explored the awareness and intention-to-use on conceptional applications of AI and BC, rather than a singular digital application or therapeutic intervention, i.e., an mHealth application. Future research on the adoption of digital health tools should incorporate a study design with follow-up survey to provide more insight into the long-term impact of the educational intervention on physicians and patients perceptions and understanding the factors that hinder or promote adherence to prevent discontinuation of their use.

## 5 Conclusion

The study emphasizes the role of professional education in bridging the gap between the evidenced clinical benefits and actual use of emerging health technologies in breast cancer care. It indicates that healthcare professionals in gynecological oncology are open to digital tools and show a sufficient degree of eHealth literacy. The adoption of emerging technologies nevertheless lags behind, often due to a lack of awareness, time and training. The study demonstrates that even brief educational interventions show the potential to increase the intention-to-use emerging, less-known technologies, suggesting that focused educational programs could significantly enhance the integration of emerging technologies into clinical practice.

## Data availability statement

The raw data supporting the conclusions of this article will be made available by the authors, without undue reservation.

## Ethics statement

This is an observational study with primary data anonymization. The Philipps-University Marburg Research Ethics Committee has confirmed that no ethical approval is required (23**-**279 ANZ).

## Author contributions

SG: Conceptualization, Data curation, Formal analysis, Investigation, Methodology, Software, Validation, Visualization, Writing – original draft, Writing – review & editing. JK: Conceptualization, Formal analysis, Investigation, Methodology, Validation, Writing – review & editing. NG: Conceptualization, Formal analysis, Investigation, Methodology, Validation, Writing – review & editing. MW: Supervision, Writing – review & editing. UW: Resources, Supervision, Writing – review & editing. ML: Project administration, Resources, Supervision, Writing – review & editing. SK: Methodology, Project administration, Resources, Supervision, Validation, Writing – review & editing.
