# Supplementary material for: Awareness and intention-to-use of digital health applications, artificial intelligence and blockchain technology in breast cancer care
Source: Front Med (Lausanne). 2024 May 2;11:1380940. doi: 10.3389/fmed.2024.1380940 (PMC11177209; doi:10.3389/fmed.2024.1380940)
Supplement: Supplementary file 1 [file Data_Sheet_1.PDF]

Einstiegsfragen

* Gibt eine erforderliche Frage an

1. Zur späteren Zusammenführung der Fragebögen benötigen wir ein Pseudonym. *

   Bitte geben Sie hierzu das **Geburtsjahr Ihres Vaters** und **Ihre aktuelle Hausnummer** an:

   z.B. 1961 - 31

2. Wie alt sind Sie? *

3. Welchem Geschlecht ordnen Sie sich zu? *

   Wählen Sie alle zutreffenden Antworten aus.

   - [ ] Männlich
   - [ ] Weiblich
   - [ ] Divers

4. Arbeiten Sie vorwiegend in der stationären oder ambulanten Versorgung? *

   Wählen Sie alle zutreffenden Antworten aus.

   - [ ] Ambulant
   - [ ] Stationär

5. In welcher Position arbeiten Sie? *

Wählen Sie alle zutreffenden Antworten aus.

- [ ] Kein medizinischer Beruf
- [ ] Nicht-ärztliches medizinisches Fachpersonal
- [ ] Medizinstudent*in
- [ ] Assistenzärzt*in
- [ ] Fachärzt*in
- [ ] Oberarzt*in
- [ ] Chefärzt*in

6. Ich kenne digitale Gesundheitsanwendungen (DiGAs) *

Wählen Sie alle zutreffenden Antworten aus.

- [ ] Ja
- [ ] Nein

7. Ich habe als ÄrztIn bereits digitale Gesundheitsanwendungen (DiGAs) verschrieben? *

Wählen Sie alle zutreffenden Antworten aus.

- [ ] Ja
- [ ] Nein

8. Ich kenne Anwendungen von Künstlicher Intelligenz. *

Wählen Sie alle zutreffenden Antworten aus.

- [ ] Ja
- [ ] Nein

9. Ich kenne Anwendungen von Künstlicher Intelligenz in der Gesundheitsversorgung *

Wählen Sie alle zutreffenden Antworten aus.

- [ ] Ja
- [ ] Nein

10. Ich kenne Anwendungen von Künstlicher Intelligenz in der Brustkrebsversorgung. *

Wählen Sie alle zutreffenden Antworten aus.

- [ ] Ja
- [ ] Nein

11. Ich würde Anwendungen von Künstlicher Intelligenz in der Brustkrebsversorgung nutzen. *

(Für diese Frage haben die Möglichkeit zwischen sieben Abstufungen zu wählen)

Markieren Sie nur ein Oval.

	1	2	3	4	5	6	7	
Trifft	◯	◯	◯	◯	◯	◯	◯	Trifft zu

12. Ich kenne Anwendungen von Blockchain Technologie. *

Wählen Sie alle zutreffenden Antworten aus.

- [ ] Ja
- [ ] Nein

13. Ich kenne Anwendungen von Blockchain Technologie in der Gesundheitsversorgung *

Wählen Sie alle zutreffenden Antworten aus.

- [ ] Ja
- [ ] Nein

14. Ich kenne Anwendungen von Blockchain Technologie in der Brustkrebsversorgung. *

Wählen Sie alle zutreffenden Antworten aus.

- [ ] Ja
- [ ] Nein

15. Ich würde Anwendungen von Blockchain Technologie in der Brustkrebsversorgung nutzen. *

(Für diese Frage haben die Möglichkeit zwischen sieben Abstufungen zu wählen)

Markieren Sie nur ein Oval.

	1	2	3	4	5	6	7	
Trifft	◯	◯	◯	◯	◯	◯	◯	Trifft zu

Bitte beantworten Sie die folgenden 8 Fragen anhand der folgenden Likert-Skala.

1) Stimmt überhaupt nicht zu

2) Stimme nicht zu

3) Unentschieden

4) Stimme zu

5) Stimmt voll und ganz zu

16. Ich weiß, wie ich Internetseiten mit hilfreichen Gesundheitsinformationen finden kann. *

Markieren Sie nur ein Oval.

	1	2	3	4	5	
Stim	◯	◯	◯	◯	◯	Stimme voll und ganz zu

17. Ich weiß, wie ich das Internet nutzen kann, um Antworten auf meine Gesundheitsfragen zu erhalten. *

Markieren Sie nur ein Oval.

	1	2	3	4	5	
Stim	◯	◯	◯	◯	◯	Stimme voll und ganz zu

18. Ich weiß, welche Seiten mit Gesundheitsinformationen im Internet verfügbar sind. *

Markieren Sie nur ein Oval.

	1	2	3	4	5	
Stim	◯	◯	◯	◯	◯	Stimme voll und ganz zu

19. Ich weiß, wo ich im Internet hilfreiche Gesundheitsinformationen finden kann. *

Markieren Sie nur ein Oval.

	1	2	3	4	5	
Stim	◯	◯	◯	◯	◯	Stimme voll und ganz zu

20. Ich weiß Gesundheitsinformationen aus dem Internet so zu nutzen, dass sie mir weiterhelfen. *

Markieren Sie nur ein Oval.

	1	2	3	4	5	
Stim	◯	◯	◯	◯	◯	Stimme voll und ganz zu

21. Ich bin in der Lage, Internetseiten mit Gesundheitsinformationen kritisch zu bewerten. *

Markieren Sie nur ein Oval.

	1	2	3	4	5	
Stim	◯	◯	◯	◯	◯	Stimme voll und ganz zu

22. Ich kann zwischen vertrauenswürdigen und fragwürdigen Internetseiten mit Gesundheitsinformationen unterscheiden. *

Markieren Sie nur ein Oval.

	1	2	3	4	5	
Stim	◯	◯	◯	◯	◯	Stimme voll und ganz zu

23. Ich fühle mich sicher darin, Informationen aus dem Internet zu nutzen, um Entscheidungen in Bezug auf meine Gesundheit zu treffen. *

Markieren Sie nur ein Oval.

	1	2	3	4	5	
Stim	◯	◯	◯	◯	◯	Stimme voll und ganz zu

Dieser Inhalt wurde nicht von Google erstellt und wird von Google auch nicht unterstützt.

Google Formulare
