# Supplementary material for: Awareness and intention-to-use of digital health applications, artificial intelligence and blockchain technology in breast cancer care
Source: Front Med (Lausanne). 2024 May 2;11:1380940. doi: 10.3389/fmed.2024.1380940 (PMC11177209; doi:10.3389/fmed.2024.1380940)
Supplement: Supplementary file 2 [file Data_Sheet_2.PDF]

Item	Factor Loading	Item-Total Correlation
GR_eHEALS_1	0.780	0.762
GR_eHEALS_2	0.898	0.793
GR_eHEALS_3	0.831	0.786
GR_eHEALS_4	0.787	0.802
GR_eHEALS_5	0.906	0.817
GR_eHEALS_6	0.794	0.795
GR_eHEALS_7	0.747	0.785
GR_eHEALS_8	0.619	0.750
Variance accounted for		68.37%
Coefficient alpha		0.928

S2: eHEALS scale reliability and exploratory factor analysis
